# Development of a novel, clinically relevant anoikis-related gene signature to forecast prognosis in patients with prostate cancer

**DOI:** 10.3389/fgene.2023.1166668

**Published:** 2023-08-30

**Authors:** Xiaolin Liu, Kunming Wang

**Affiliations:** Department of Urology, Sunshine Union Hospital, Weifang, Shandong, China

**Keywords:** prostate cancer, anoikis, tumor microenvironment, gene signature, prognosis

## Abstract

**Introduction:** Anoikis is a specific form of programmed cell death and is related to prostate cancer (PC) metastasis. This study aimed to develop a reliable anoikis-related gene signature to accurately forecast PC prognosis.

**Methods:** Based on anoikis-related genes and The Cancer Genome Atlas (TCGA) data, anoikis-related molecular subtypes were identified, and their differences in disease-free survival (DFS), stemness, clinical features, and immune infiltration patterns were compared. Differential expression analysis of the two subtypes and weighted gene co-expression network analysis (WGCNA) were employed to identify clinically relevant anoikis-related differentially expressed genes (DEGs) between subtypes, which were then selected to construct a prognostic signature. The clinical utility of the signature was verified using the validation datasets GSE116918 and GSE46602. A nomogram was established to predict patient survival. Finally, differentially enriched hallmark gene sets were revealed between the different risk groups.

**Results:** Two anoikis-related molecular subtypes were identified, and cluster 1 had poor prognosis, higher stemness, advanced clinical features, and differential immune cell infiltration. Next, 13 clinically relevant anoikis-related DEGs were identified, and five of them (*CKS2*, *CDC20*, *FMOD*, *CD38*, and *MSMB*) were selected to build a prognostic signature. This gene signature had a high prognostic value. A nomogram that combined Gleason score, T stage, and risk score could accurately predict patient survival. Furthermore, gene sets closely related with DNA repair were differentially expressed in the different risk groups.

**Conclusion:** A novel, clinically relevant five-anoikis-related gene signature was a powerful prognostic biomarker for PC.

## 1 Introduction

Prostate cancer (PC) is a common solid tumor in men worldwide, and most PCs are diagnosed as prostate adenocarcinoma (PRAD). Most patients with PC are asymptomatic in the early stages; however, 17%–33% of patients undergo biochemical recurrence after initial radical prostatectomy, and 20%–30% of individuals develop advanced or metastatic disease (Ward et al., 2003; Chandrasekar et al., 2015; Matsumoto et al., 2018). Androgen deprivation therapy (ADT) is the primary management strategy for advanced or metastatic PC; however, cancer recurrence often occurs, and this malignancy is likely to progress to castration-resistant prostate cancer (CRPC) within 2–3 years after ADT treatment (Chandrasekar et al., 2015; Achard et al., 2022). CRPC is associated with very poor prognosis, and its treatment remains a serious clinical challenge (Ruiz de Porras et al., 2021; Leith et al., 2022). Considering that PC is highly heterogeneous in terms of molecular alterations and variable clinical courses (Inamura, 2018), subtyping provides a novel perspective on the molecular pathologies of cancer and allows the implementation of personalized therapies. Therefore, it is necessary to explore cancer subtypes and develop a reliable prognostic signature for risk stratification of patients with PC.

Anoikis is a special type of programmed cell death that results from the loss of cell adhesion or inappropriate cell adhesion (Kakavandi et al., 2018). Accumulating evidence has revealed that anoikis can regulate the survival of tumor cells after they are detached from the extracellular matrix (ECM) and plays a pivotal role in preventing cancer metastasis (Buchheit et al., 2014; Raeisi et al., 2022). During PC progression, ECM undergoes intense changes and plays a key role in cytoskeleton rearrangement (Rennebeck et al., 2005a). In the process of epithelial–mesenchymal transition (EMT), tumor epithelial cells characterized by mesenchymal features can migrate to the stroma, underlying the activation of survival pathways (Romashkova and Makarov, 1999). Normal epithelial cells undergo anoikis when detached from ECM, while tumor cells are associated with anoikis resistance (Rennebeck et al., 2005a). The generation of anoikis resistance is considered an important condition for tumor metastasis because anchorage-independent growth of tumor cells is an important feature of various human cancers, including PC (Rennebeck et al., 2005b; Kim et al., 2012; Lee et al., 2021). It has been reported that, in PC cell lines, the expression of αvβ3 integrin was vastly reduced, which contributes to the migratory phenotype of tumor cells (Zheng et al., 1999). Moreover, targeting anoikis resistance has become a therapeutic promise for metastatic PC via prevention of tumor metastasis (Sakamoto and Kyprianou, 2010). Some recent studies have focused on identifying key anoikis-related genes to reveal the possible mechanisms of tumor progression and establish anoikis-related gene signatures for cancer prognosis (Chi et al., 2022; Zhao et al., 2022). However, the crucial anoikis-related genes associated with PC prognosis have not yet been fully elucidated.

Herein, we identified anoikis-related molecular subtypes based on anoikis-related genes extracted from the literature reported by Sun et al. (Sakamoto and Kyprianou, 2010) and The Cancer Genome Atlas (TCGA)-PRAD dataset and analyzed their differences in disease-free survival (DFS), stemness index (si), clinical features, and immune infiltration patterns. Differential expression analysis of the two subtypes and weighted gene co-expression network analysis (WGCNA) were utilized to investigate clinically relevant anoikis-related differentially expressed genes (DEGs). Furthermore, a prognostic signature was constructed using key anoikis-related genes, and its clinical utility was validated using the independent validation datasets GSE116918 and GSE46602. A nomogram containing the risk score of the prognostic signature and other independent clinical features was created to predict patient survival. Finally, the differentially enriched hallmark gene sets between the different risk groups were revealed. Our efforts were devoted to developing a novel signature based on anoikis-related genes that could accurately predict PC, thereby improving treatment options.

## 2 Methods

### 2.1 Data acquisition and preprocessing

The gene expression RNA-seq data [log2(fpkm + 1)] in the Genomic Data Commons (GDC) TCGA-PRAD was downloaded from the UCSC Xena platform (Goldman et al., 2019). The samples with tissue number “-01A” and DFS information were selected, and, finally, 480 PRAD samples were included. The corresponding clinical information for these samples, including age, nonsynonymous tumor mutational burden (TMB), Gleason score, primary tumor laterality, new neoplasm event post initial therapy indicator, overall survival time and status, N stage, and T stage, was obtained from the cBioportal website (http://www.cbioportal.org/), The gene expression data (GSE116918 (Jain et al., 2018) and GSE46602 (Mortensen et al., 2015) were downloaded from NCBI Gene Expression Omnibus (GEO) (Barrett et al., 2005). A total of 247 PC samples with DFS information were retained from the GSE116918 dataset and 36 PC samples from the GSE46602 dataset. The raw expression profiles were downloaded and subjected to preprocessing, normalization, and log2 transformation. Then, the probe number was matched to the gene symbol according to the platform annotation file. Probes without matching gene symbols were removed. If multiple probes corresponded to the same gene symbol, the mean expression value of the identified gene was calculated.

### 2.2 Prediction of anoikis-related molecular subtypes

We extracted 27 anoikis-related genes from the literature published by Sun et al., (2022) and obtained their expression values in 480 samples from the TCGA-PRAD dataset. Anoikis-related molecular subtypes of PC samples were identified using ConsensusClusterPlus (version 1.54.0) (Wilkerson and Hayes, 2010) in R 3.6.1. The parameters were set as follows: cluster algorithm: PAM; correlation method: Spearman; feature subsampling proportion: 1; and item subsampling proportion: 0.85.

### 2.3 Prognostic analysis for different subtypes

Based on the DFS information for each sample in the subtypes, Kaplan–Meier (K–M) survival analyses for different subtypes were performed, followed by analysis of their differences using the logrank test.

### 2.4 Comparison of clinical features between subtypes

The clinical information relating to different subtypes, including age, nonsynonymous TMB, Gleason score, primary tumor laterality, new neoplasm event post initial therapy indicator, overall survival time and status, N stage, and T stage was sorted. For factorial variables, the chi-square test was utilized to compare differences between different subtypes. For continuous variables, the Wilcoxon test was applied to analyze the differences between subtypes.

### 2.5 Analysis of si differences between subtypes

The si of a sample can be calculated using a one-class logistic regression (OCLR) machine learning algorithm based on mRNA expression or DNA methylation (Malta et al., 2018). In this study, we extracted the si of TCGA-PRAD samples based on mRNA expression (mRNAsi). The mRNAsi of the consensus cluster subtypes was compared using the Wilcoxon test.

### 2.6 Comparison of immune infiltration between subtypes

To observe differences in the immune microenvironment between subtypes, the proportion of 22 types of immune cells in the TCGA-PRAD samples was calculated using the CIBERSORT algorithm (Kawada et al., 2021). Differences between subtypes were analyzed using the Wilcoxon test. The stromal, immune, and ESTIMATE scores of the TCGA-PRAD samples were assessed using the ESTIMATE algorithm (Yoshihara et al., 2013), and the differences between subtypes were also evaluated using the Wilcoxon test. Furthermore, we extracted the expression levels of key immune checkpoint genes from the TCGA-PRAD samples and analyzed the differences between subtypes using a *t*-test.

### 2.7 Identification of DEGs between subtypes and functional enrichment analysis

Based on the TCGA-PRAD data, DEGs between subtypes were screened using the limma package (version 3.1.3) (Smyth, 2005). The threshold value for DEG screening was set as |log fold change| > 0.585 and the Benjamini–Hochberg (BH) procedure-adjusted *p*-value (adj.p.value) was < 0.05.

Functional enrichment analysis was performed using DAVID (version 6.8) (Sherman et al., 2022). The significantly enriched Gene Ontology (GO) (Ashburner et al., 2000) terms and Kyoto Encyclopedia of Genes and Genomes (KEGG) (Kanehisa and Goto, 2000) pathways by DEGs were obtained. A *p*-value <0.05 and gene count ≥2 were set as the cutoff values.

### 2.8 WGCNA for selecting clinically relevant genes

WGCNA was performed to screen clinically significant modules and explore genes closely related to clinical traits. First, we calculated the degree of changes in gene expression in the TCGA-PRAD samples and selected the top 75% genes with the largest variation to construct a weighted gene co-expression network using the R WGCNA package (version 1.61) (Langfelder and Horvath, 2008). In the WGCNA algorithm, the soft-thresholding power that could make gene connections conforming to a scale-free network was selected. Next, using clustering and dynamic pruning methods, highly interconnected genes were clustered into modules using the following parameters: minModuleSize = 50 and MEDissThres = 0.3. By evaluating the correlation between gene modules and clinical traits (including age, nonsynonymous TMB, Gleason score, primary tumor laterality, new neoplasm event post initial therapy indicator, overall survival time and status, N stage, and T stage), clinically significant modules were obtained with a cutoff value of correlation coefficient > 0.5 and *p*-value < 0.05. The genes in clinically significant WGCNA modules were considered clinically relevant.

### 2.9 Construction and validation of the prognostic signature

The intersection analysis of DEGs between subtypes and clinically relevant genes was carried out to obtain overlapping DEGs related to both anoikis and clinical features of PC. By combining the expression level of overlapping DEGs with the DFS information for each sample in the TCGA-PRAD training dataset, univariate Cox regression analysis was performed using the survival package (version 2.41-1) (Wang et al., 2016) in R 3.6.1. The genes with *p*-value < 0.05 were considered prognosis-related and were selected for further LASSO Cox regression analysis (Tibshirani, 1997) using the glmnet package (version 2.0-18) (Friedman et al., 2009). The optimal combination of prognosis-related genes was selected using 20-fold cross-validation. The prognostic signature was then constructed to calculate the risk score of the samples, using the following formula: risk score = ∑β_gene_ × Exp_gene_, where β_gene_ represented the LASSO Cox regression coefficient of each gene, and Exp_gene_ referred to the gene expression in each sample.

To confirm the accuracy of the prognostic signature, the risk scores of each sample in the TCGA-PRAD training dataset and the GSE116918 and GSE46602 validation datasets were calculated. The samples in the TCGA-PRAD, GSE116918, and GSE46602 cohorts were divided into high- and low-risk groups according to the median value of the risk score. The K–M survival curves of the two risk groups were plotted using the survival package (version 2.41-1) in R3.6.1. The performance of the prognostic signature in evaluating the 1-, 3-, and 5-year survival probabilities was measured using receiver operating characteristic (ROC) analysis.

### 2.10 Establishment and validation of nomogram

To investigate whether the risk score was an independent prognostic factor for patients with PC, univariate and multivariate Cox regression analyses were conducted, combined with other clinical variables, including age, nonsynonymous TMB, Gleason score, N stage, and T stage. Independent prognostic factors were obtained with a *p*-value < 0.05. These prognostic factors were then combined to establish a nomogram to assess the probabilities of 1-, 3-, and 5-year survival. Calibration curves were plotted to assess the validity of the nomogram.

### 2.11 Gene set enrichment analysis (GSEA)

Using h.all.v7.4.symbols.gmt from MSigDB v7.1 (Liberzon et al., 2011) as the enriched background, based on the gene expresssion value of in the TCGA-PRAD samples, GSEA was conducted to determine the enriched hallmark gene sets between the different risk groups using the R package clusterProfiler (version:3.8.1) (Yu et al., 2012). The BH procedure-adjusted *p*-value (adj.p.value) < 0.05 was used as the cutoff value for screening the significantly enriched hallmark gene sets.

### 2.12 Validation of prognostic signature in metastatic cohort

The dataset of GSE211448 was downloaded from NCBI-GEO database, which contained nine metastatic PC tissues and three primary PC samples. The expression levels of five genes (CKS2, CDC20, FMOD, CD38 and MSMB) in prognostic signature were extracted, followed by Risk score calculation. We employed ggplot 2 package to compare the risk score between metastasis group and primary group based on *t*-test.

In parallel, five pairs of clinical samples were collected from metastatic PC patients and primary PC patients with informed consent, and this study was approved by the Committee on Medical Ethics of the Sunshine Union Hospital of Weifang. The expressions of five signature genes were detected by real time PCR assay. The difference between groups was compared with *t*-test. Statistical significance was considered as *p* < 0.05.

## 3 Results

### 3.1 Two anoikis-related molecular subtypes were clustered, which had different DFS, sis, and clinical features

**FIGURE 1:**
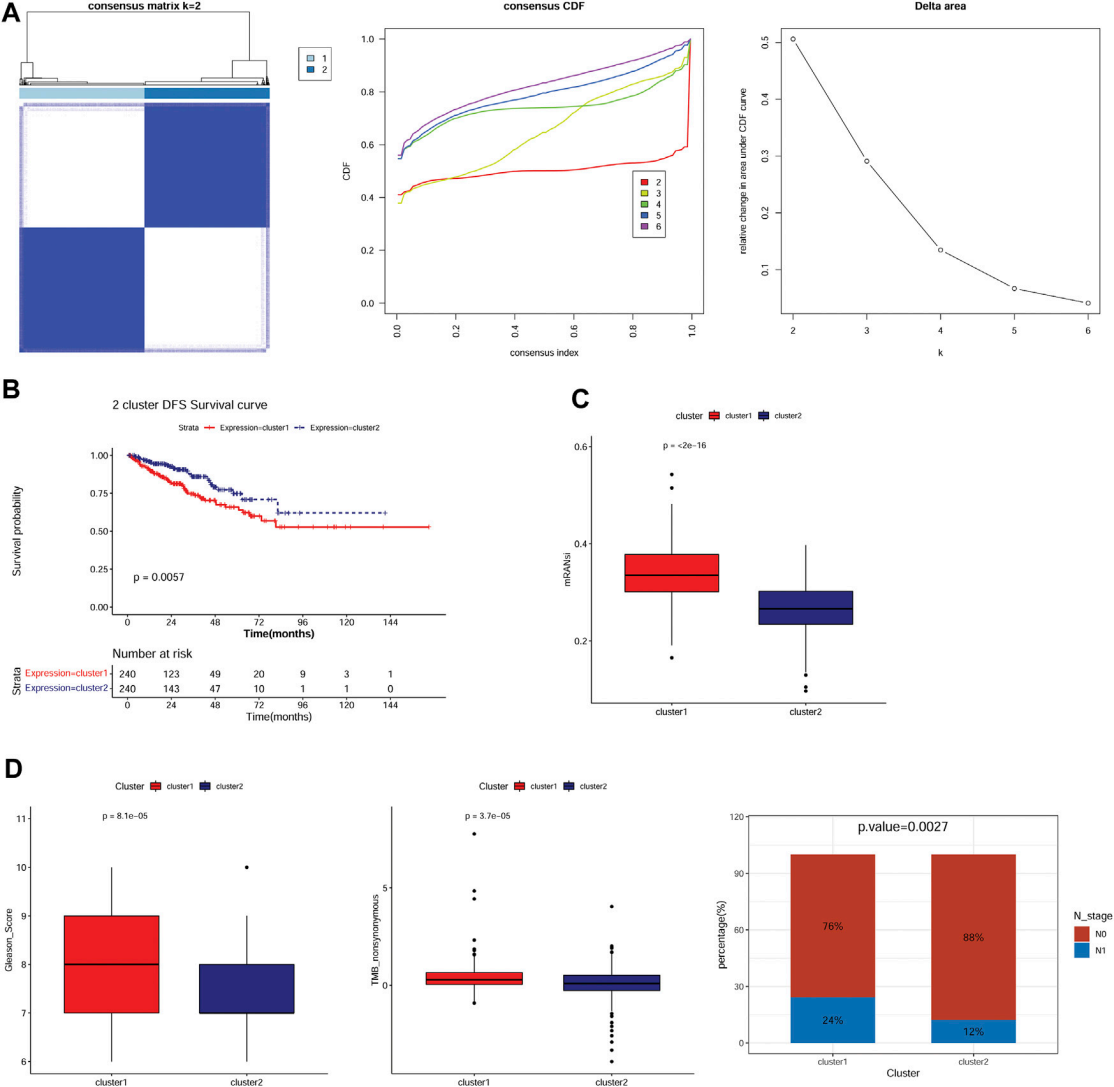
Two anoikis-related molecular subtypes were identified, which had different survival, mRNAsi, and clinical features. **(A)**: Subtype clustering heatmap, cumulative distribution function (CDF) distribution curve, and Delta area line graph. **(B)**: Kaplan–Meier (K–M) survival curves for samples in two subtypes. **(C)**: The stemness signature of two subtypes. **(D)**: The distribution of clinical features including Gleason score, TMB nonsynonymous, and N stage in the two subtypes.

Based on the expression patterns of 27 anoikis-related genes in the TCGA-PRAD samples, two consensus cluster subtypes (clusters 1 and 2) were identified (Figure 1A). To determine whether patient prognosis differed in the subtype clusters, we performed K–M survival analysis. As illustrated in Figure 1B, the prognosis of cluster 1 patients was remarkably poorer than that of cluster 2 patients. We analyzed the stemness signature of the subtypes and observed a higher mRNAsi in cluster 1 than in cluster 2 (Figure 1C). Further analysis of the clinical features showed significant differences in the Gleason score, nonsynonymous TMB, and N stage between the two subtypes (Table 1). Compared with cluster 2, cluster 1 showed a higher Gleason score, nonsynonymous TMB, and N1 stage percentage (Figure 1D), which might be associated with the poor prognosis of cluster 1.

**TABLE 1:** The clinical characteristics of two anoikis-related molecular subtypes of prostate cancer.

Characteristics	cluster1 (N = 240)	cluster2 (N = 240)	Total (N = 480)	*p*-value
Age				0.32
Mean ± SD	61.40 ± 6.83	60.66 ± 6.86	61.03 ± 6.85	
Median[min-max]	62.00[44.00,78.00]	61.00[41.00,75.00]	61.00[41.00,78.00]	
Gleason score				**8.10E-05**
Mean ± SD	7.79 ± 1.05	7.42 ± 0.93	7.61 ± 1.01	
Median[min-max]	8.00[6.00,10.00]	7.00[6.00,10.00]	7.00[6.00,10.00]	
Primary tumor laterality				0.24
Bilateral	208(44.07%)	212(44.92%)	420(88.98%)	
Left	7(1.48%)	10(2.12%)	17(3.60%)	
Right	22(4.66%)	13(2.75%)	35(7.42%)	
TMB nonsynonymous				**3.70E-05**
Mean ± SD	2.47 ± 14.13	1.29 ± 1.21	1.88 ± 10.03	
Median[min-max]	1.22[0.53,217.63]	1.07[0.07,16.47]	1.17[0.07,217.63]	
New neoplasm event post initial therapy indicator				0.27
NO	141(45.48%)	104(33.55%)	245(79.03%)	
YES	43(13.87%)	22(7.10%)	65(20.97%)	
Overall survival time				0.81
Mean ± SD	36.41 ± 28.83	34.39 ± 21.89	35.40 ± 25.59	
Median[min-max]	30.09[0.76,165.05]	30.59[0.89,141.10]	30.42[0.76,165.05]	
Overall survival status				0.13
DECEASED	4(0.83%)	0(0%)	4(0.83%)	
LIVING	236(49.17%)	240(50.00%)	476(99.17%)	
N stage				**2.70E-03**
N0	156(38.14%)	178(43.52%)	334(81.66%)	
N1	50(12.22%)	25(6.11%)	75(18.34%)	
T stage				0.11
T2	81(17.09%)	100(21.10%)	181(38.19%)	
T3	152(32.07%)	132(27.85%)	284(59.92%)	
T4	6(1.27%)	3(0.63%)	9(1.90%)	

The bold values indicates means the *p* value < 0.05

### 3.2 Different immune infiltration patterns between subtypes

**FIGURE 2:**
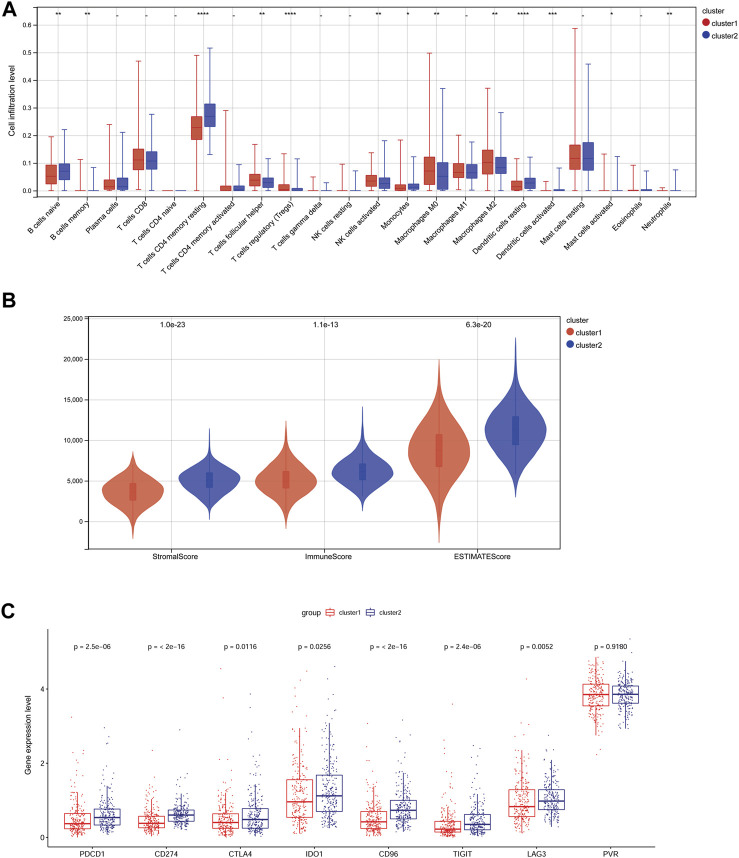
Differences in immune infiltration patterns in different subtypes. **(A)**: The infiltration proportion of 22 kinds of immune cells in two subtypes. * *p* < 0.05, ** *p* < 0.01, *** *p* < 0.001, **** *p* < 0.0001. **(B)**: Differences in the stromal score, immune score, and ESTIMATE score of two subtypes. **(C)**: The expression of immune checkpoint genes in the two subtypes.

To compare the immune microenvironment of the different subtypes, the immune infiltration patterns of the two subtypes were analyzed using the CIBERSORT algorithm. The results suggested that the proportion of 13 immune cell types, including B cells naïve, T cells CD4 memory resting, and M0 and M2 macrophages, was significantly different between the subtypes (Figure 2A). The stromal, immune, and ESTIMATE scores of the TCGA-PRAD samples in cluster 1 were significantly lower than those in cluster 2 (Figure 2B). Furthermore, except for *PVR*, the expression levels of immune checkpoint genes, including *PDCD1* (*PD-1*), *CTLA4*, *IDO1*, *CD96*, *CD274* (*PD-L1*), *LAG3*, and *TIGIT*, were significantly lower in cluster 1 than those in cluster 2 (Figure 2C).

### 3.3 DEG identification and functional enrichment analysis

**FIGURE 3:**
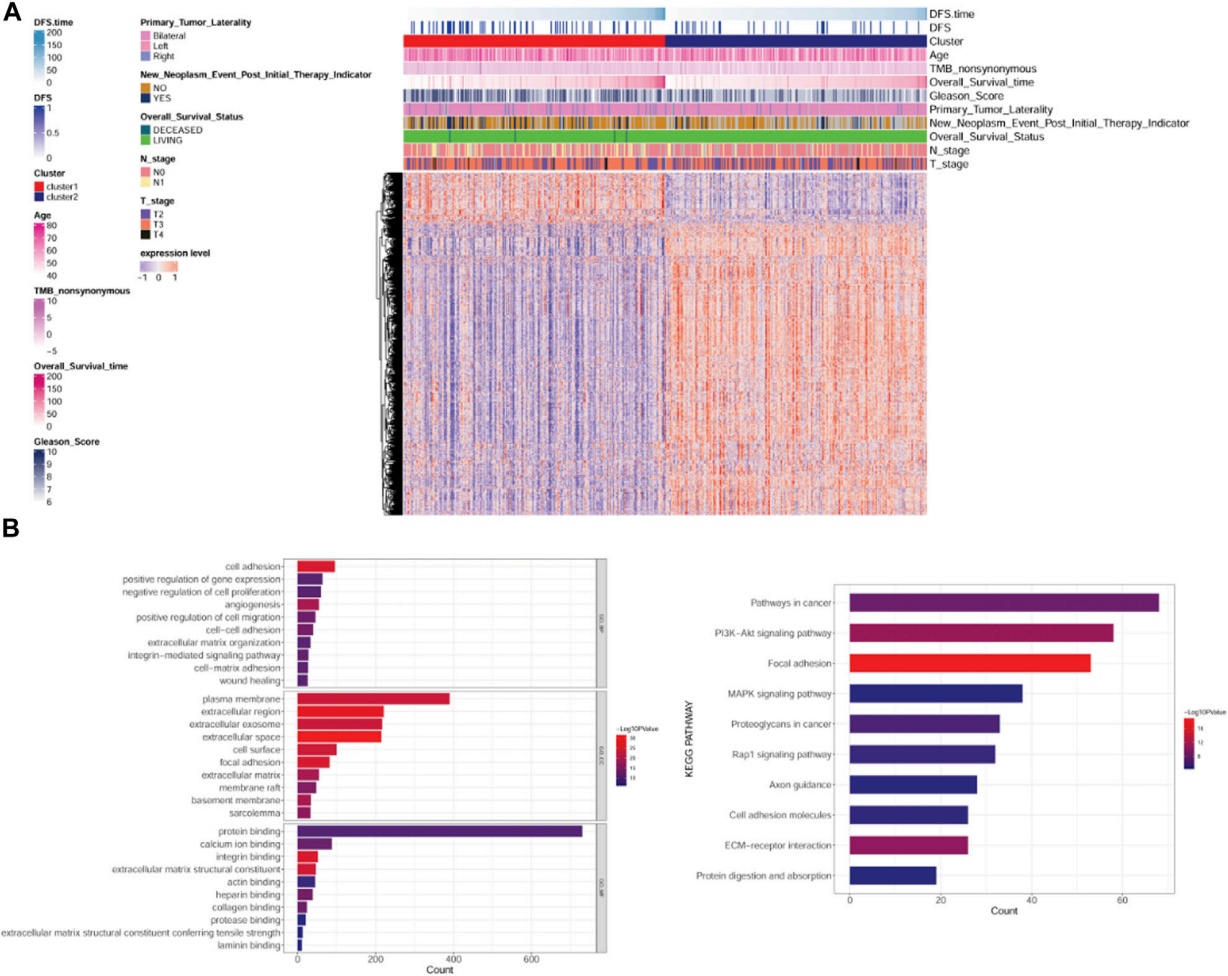
Analysis of differentially expressed genes (DEGs) between subtypes and their functional enrichment analysis. **(A)**: Heatmap of DEGs between subtypes. **(B)**: GO and KEGG pathway enrichment results. GO: Gene Ontology; KEGG: Kyoto Encyclopedia of Genes and Genomes.

Based on the cutoff value, 163 up- and 918 downregulated DEGs were identified between clusters 1 and 2. A heatmap showed that the samples in two subtypes could be clearly distinguished according to the expression patterns of the DEGs (Figure 3A). DEGs were remarkably enriched in 562 GO biological process (BP) terms (such as cell adhesion), 102 GO cellular component (CC) terms (such as extracellular region), 98 GO molecular function (MF) terms (such as ECM structural constituent), and 55 KEGG pathways (such as focal adhesion). The top 10 GO terms or pathways are shown in Figure 3B.

### 3.4 Identification of clinically relevant genes using WGCNA

**FIGURE 4:**
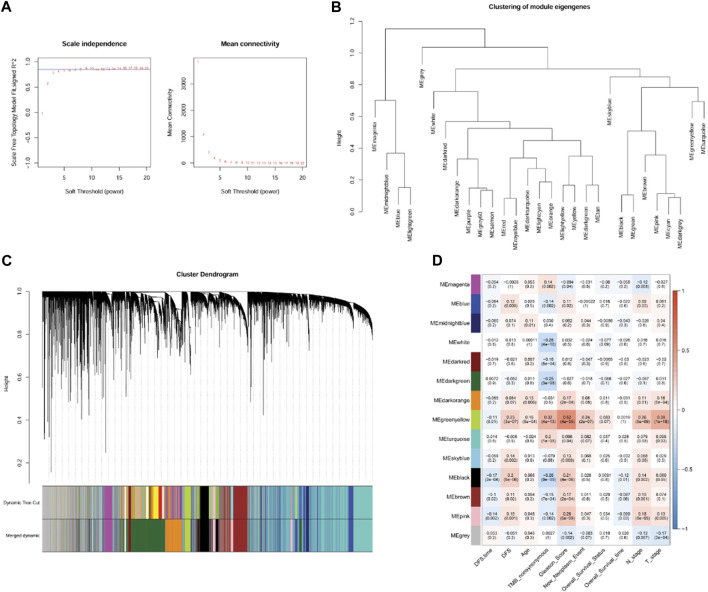
Co-expression network analysis by weighted gene co-expression network analysis (WGCNA). **(A)**: Analysis of network topology for various soft-threshold powers. **(B)**: Module clustering result. **(C)**: Results of gene module merging based on clustering and dynamic pruning methods. Each vertical line indicates a gene and each branch represents an expression module of highly interconnected genes. Below the dendrogram, different modules are given different colors. Gray indicated that genes are outside all modules. **(D)**: The correlations of gene modules with multiple clinical features.

According to the expression value of the top 75% genes with the largest variation in the PRAD samples, WGCNA was conducted, and the soft-thresholding power of 8 was selected (Figure 4A). Using clustering and dynamic pruning methods, highly interconnected genes were clustered into modules, and the modules with correlation coefficients greater than 0.7 were merged. Finally, 13 modules were identified (Figures 4B, C). By analyzing the correlation between module genes and clinical phenotypes of the PC samples, the green-yellow module containing 523 genes had the largest correlation coefficient with the Gleason score and significant correlations with all other clinical features (Figure 4D); therefore, the genes in this module were regarded as clinically relevant.

### 3.5 The prognostic signature was constructed based on five clinically relevant anoikis-related genes

**FIGURE 5:**
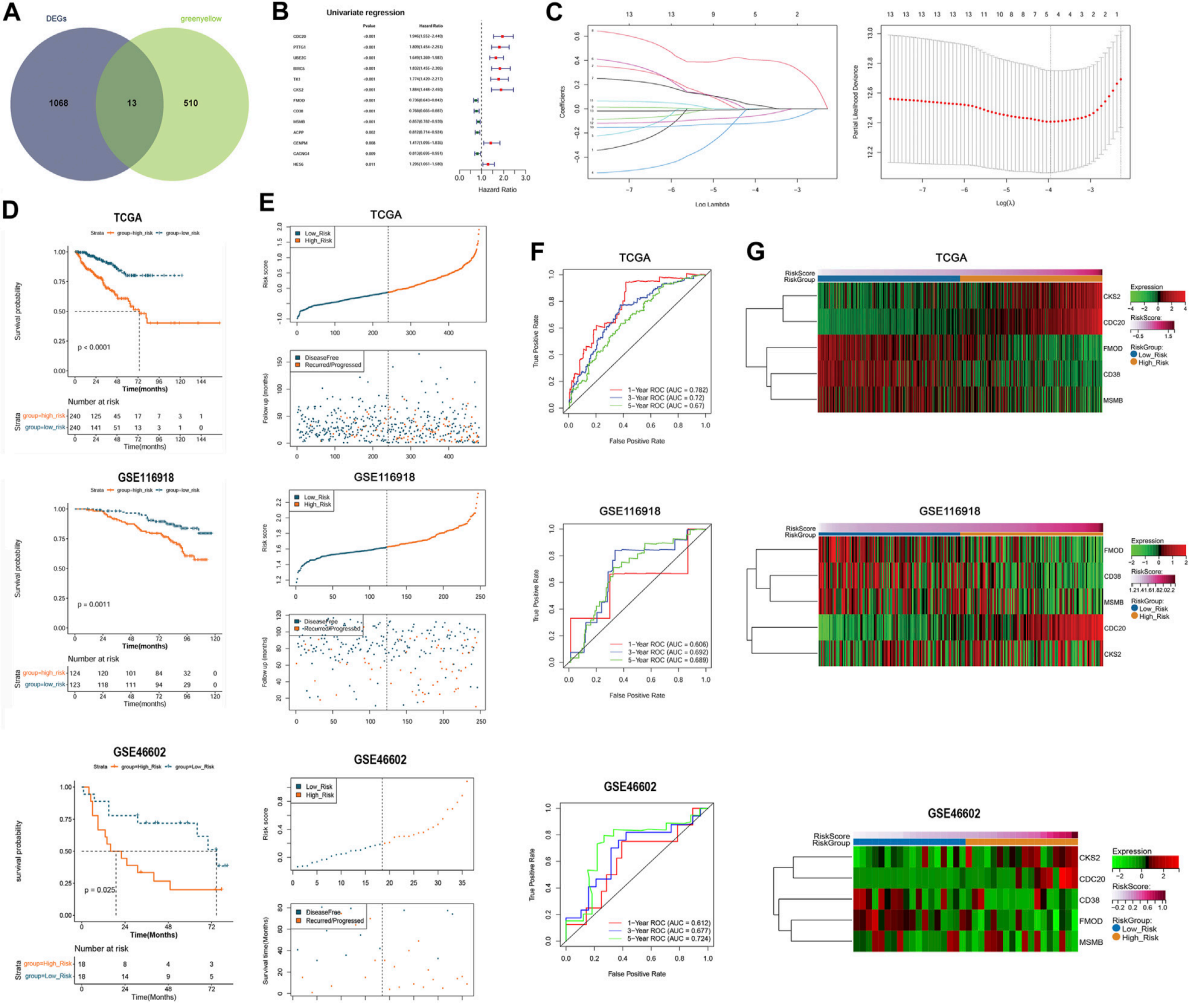
Construction and validation of the prognostic signature that was established by five anoikis-related genes. **(A)**: Venn plot showed the overlapping DEGs related to both anoikis and clinical features of prostate cancer. **(B)**: Univariate Cox regression analysis showed that the overlapping DEGs were all significantly correlated with DFS of patients. **(C)**: The LASSO coefficient spectrum of the prognostic DEGs and optimized lambda determined in the LASSO regression model. **(D)**: Kaplan–Meier (K–M) survival curves showed the survival differences between the two risk groups based on TCGA-PRAD training dataset and GSE116918 validation dataset. **(E)**: The scatterplots showed the distribution of the risk score and recurred/progressed time of patients based on TCGA-PRAD training dataset and GSE116918 validation dataset. **(F)**: ROC curves revealed the predictive performance of the gene signature in predicting 1-, 3-, and 5-year survival probabilities based on TCGA-PRAD training dataset and GSE116918 validation dataset. **(G)**: Heatmap showed significant differences in the expression of the five model genes between the high- and low-risk samples in the TCGA-PRAD training dataset and GSE116918 validation dataset.

We conducted an intersection analysis of DEGs between the subtypes and clinically relevant genes in the green-yellow module and identified 13 overlapping DEGs related to both anoikis and clinical features of PC (Figure 5A). According to the expression value of overlapping DEGs and the DFS information in each sample, univariate Cox regression analysis indicated that all these genes were significantly correlated with DFS (Figure 5B). The optimal gene combination was screened using LASSO Cox regression analysis (Figure 5C), ultimately leading to a prognostic signature constructed using five genes: cyclin-dependent kinases regulatory subunit 2 (*CKS2*), cell division cycle 20 (*CDC20*), fibromodulin (*FMOD*), CD38 molecule (*CD38*), and microseminoprotein beta (*MSMB*). Based on the LASSO Cox regression coefficient of the five model genes and their expression values, the risk score of each sample in the TCGA-PRAD, GSE116918, and GSE46602 datasets was calculated. The samples in the three datasets were then divided into high- and low-risk groups. The K–M survival curves revealed shorter DFS in the high-risk than in the low-risk group (Figure 5D). Moreover, the samples in the three datasets were ranked according to their risk score, and samples with high-risk scores tended to be recurred or progressed (Figure 5E). Furthermore, the areas under the ROC curve (AUC) of the prognostic signature for evaluating the 1-, 3-, and 5-year survival probabilities of the TCGA-PRAD samples were 0.782, 0.72, and 0.67, respectively, and those in the GSE62452 samples were 0.606, 0.692, and 0.689, respectively. The favorable prognostic value was also determined in the GSE46602 cohort with 1-, 3-, and 5-year AUCs of 0.612, 0.677, and 0.724, respectively (Figure 5F). In addition, a heatmap revealed the differential expression patterns of the five model genes between the high- and low-risk groups in the three cohorts. The expression of *CKS2* and *CDC20* was higher in the high-risk than in the low-risk group, whereas that of *FMOD*, *CD38*, and *MSMB* was significantly upregulated in the low-risk samples compared with the high-risk group (Figure 5G).

### 3.6 The prognostic signature had independent prognostic value and a nomogram was established

**FIGURE 6:**
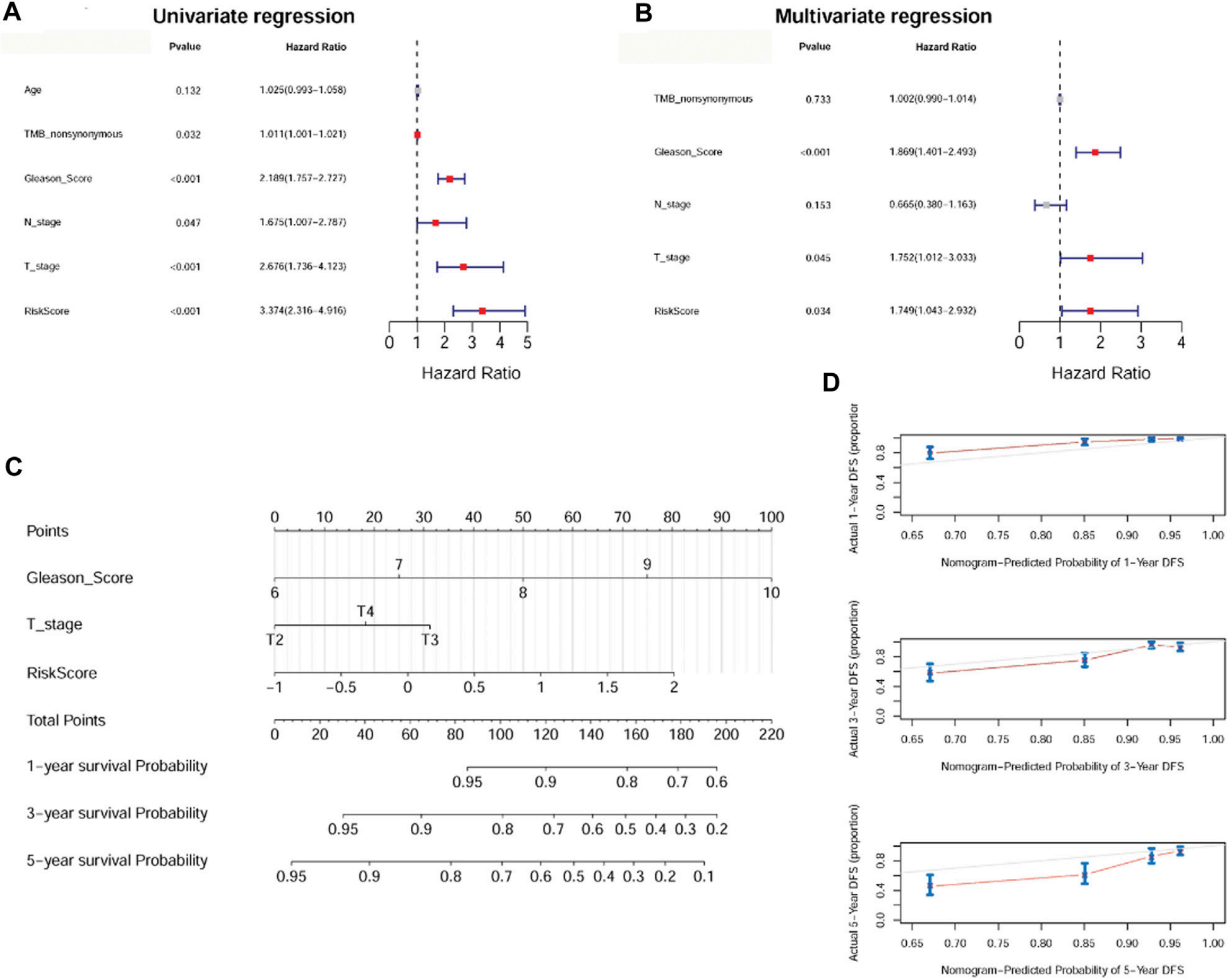
The anoikis-related gene signature was an independent prognostic factor and a nomogram was established. **(A)**: Univariate Cox regression analysis shows the correlation between survival and risk score of the anoikis-related gene signature and various clinicopathological features. **(B)**: Multivariate Cox regression analysis showed that Gleason score, T stage, and risk score were independent prognostic factors. **(C)**: A nomogram was constructed for estimating the 1-, 3-, and 5-year survival probabilities of patients. **(D)**: The calibration curves showed the concordance of the prediction probability and actual probability of 1-, 3-, and 5-year survival.

To investigate prognostic factors for PC (including risk score, age, nonsynonymous TMB, Gleason score, N stage, and T stage), we conducted univariate and multivariate Cox regression analyses. Univariate analysis indicated that risk score, nonsynonymous TMB, Gleason score, N stage, and T stage were related to DFS (*p* < 0.05, Figure 6A). Multivariate analysis revealed that risk score, Gleason score, and T stage were independent prognostic factors for patients with PC (*p* < 0.05, Figure 6B). A nomogram was then established using these independent prognostic factors, which could accurately predict the 1-, 3-, and 5-year survival probabilities (Figure 6C). The calibration curves displayed satisfactory overlap in the predictive and actual 1-, 3-, and 5-year survival probabilities, indicating the validity of our constructed nomogram (Figure 6D).

### 3.7 Analysis of differentially enriched hallmark gene sets between different risk groups

**FIGURE 7:**
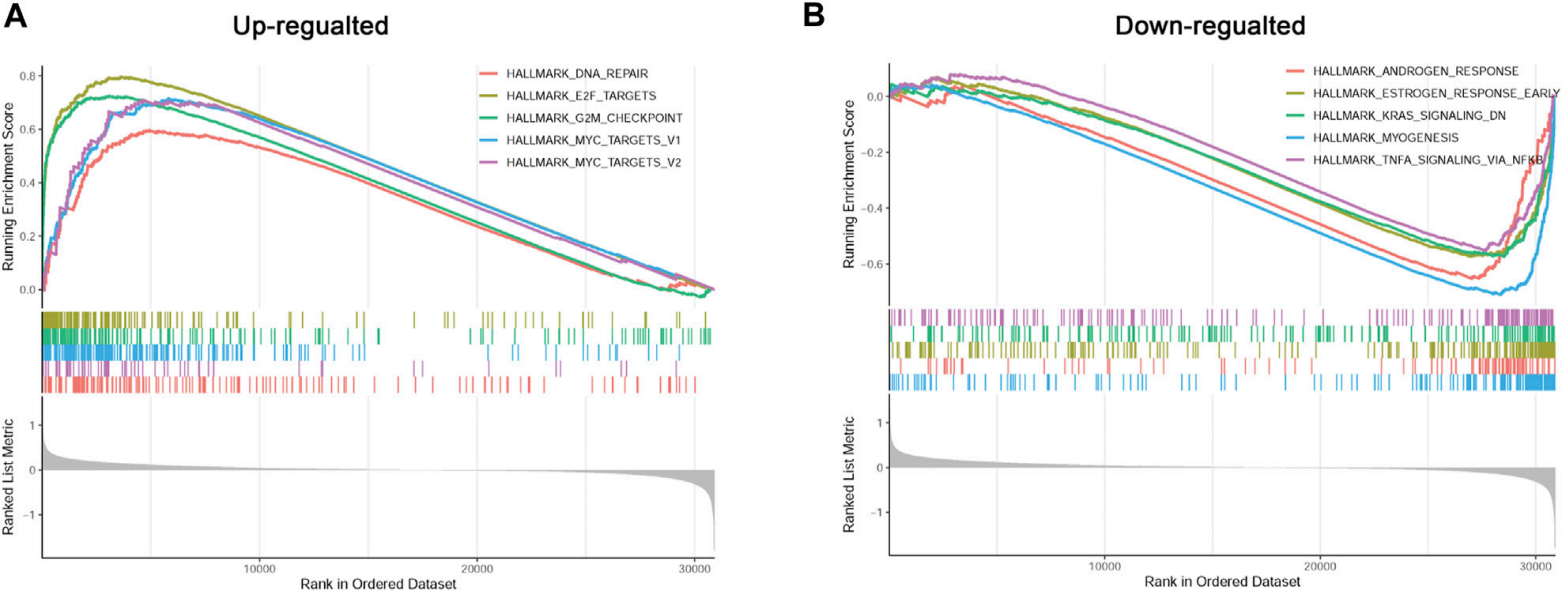
Gene set enrichment analysis (GSEA) of differentially enriched hallmark gene sets between different risk groups. **(A)**: The up-regulated hallmark gene sets in the high-risk group. **(B)**: The down-regulated hallmark gene sets in the high-risk group.

To better understand the possible regulatory mechanism underlying PC in the different subtypes, we conducted GSEA to identify the functions of enriched hallmark gene sets derived from the different risk groups. A total of 10 upregulated hallmark gene sets, such as DNA repair, and 18 downregulated hallmark gene sets, such as androgen response, with *p*-value < 0.05 were acquired from the high-risk compared with the low-risk group. The top five enriched hallmark gene sets are shown in Figure 7.

### 3.8 Prognostic signature for metastatic PC

**FIGURE 8:**
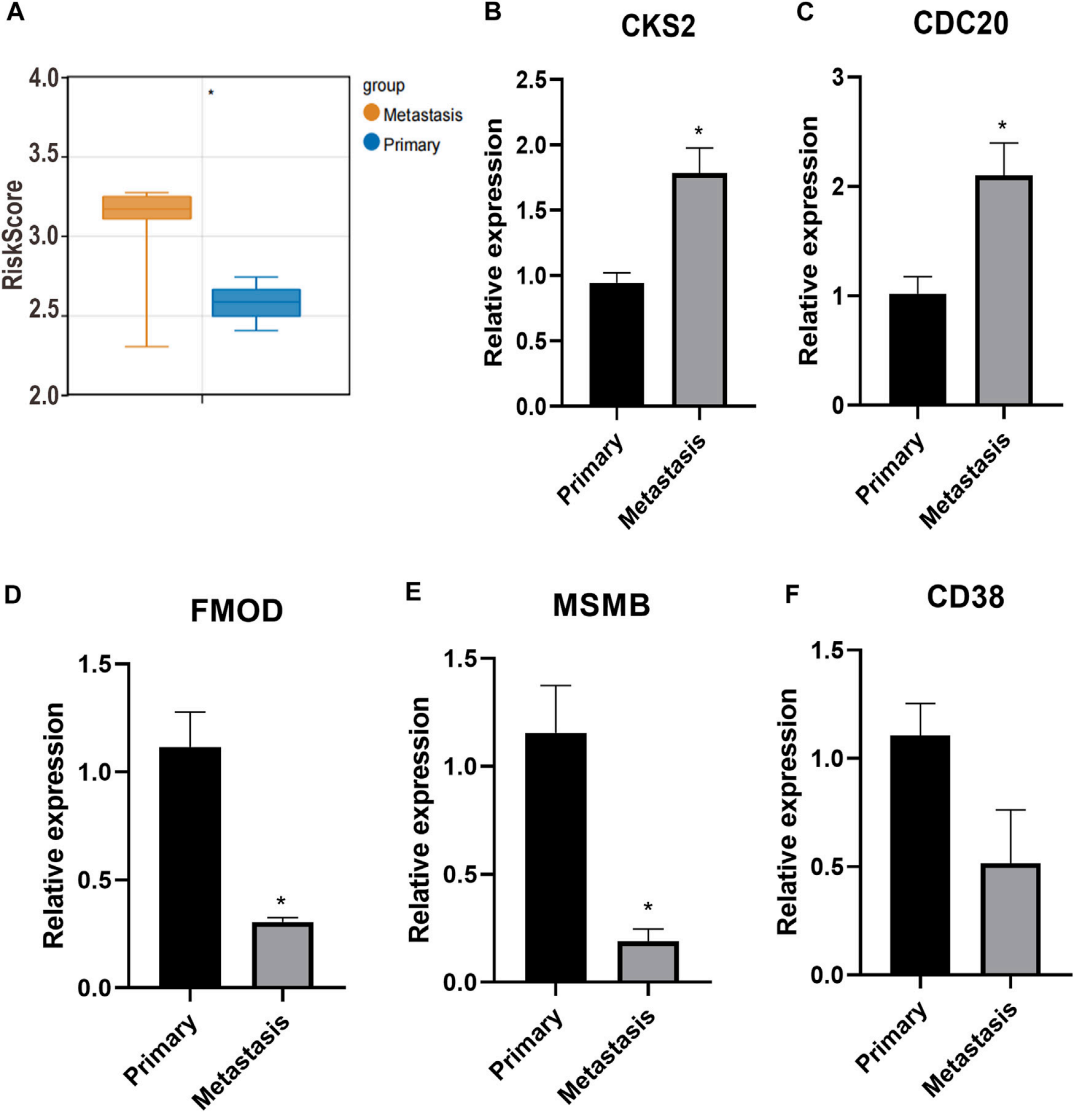
Prognostic signature for metastatic PC. **(A)**: The mean risk score between metastasis group and primary group. **(B–F)**: CKS2, CDC20, FMOD, MSMB and CD38 expression detected by RT-qPCR. *p < 0.05.

The risk score of each sample in GSE211448 cohort was calculated based on the formula mentioned above. As depicted in Figure 8A, the mean risk score was significantly higher in the metastasis group compared to primary group (*p* < 0.05), indicating the predictive value of the 5-gene signature in PC metastasis. Besides, RT-qPCR assay indicated that CKS2 and CDC20 were overexpressed in metastatic PC samples compared with primary ones, while FMOD and MSMB were de-expressed (*p* < 0.05, Figures 8B–E). No significant changes of CD38 were observed (*p* > 0.05, Figure 8F), which could be verified in a large sample size.

## 4 Discussion

PC poses a significant public health burden, and advanced or metastatic disease has a poor prognosis. The current risk evaluation and management strategies for PC mainly refer to clinical indicators of patients, such as serum prostate-specific antigen (PSA) and Gleason score (Huber et al., 2015). However, such indicators are not sufficient to accurately evaluate disease risk and treatment response (Harlan et al., 2003; Huber et al., 2015). Therefore, it is necessary to explore additional biomarkers for predicting PC risk and for assessing prognosis.

Herein, to the best of our knowledge, we first identified two anoikis-related molecular subtypes of PC with different DFS, mRNAsi, clinical features, and immune infiltration patterns. The DEGs between subtypes were significantly enriched in functions and pathways associated with focal adhesion. By combining with the WGCNA results, we screened 13 overlapping DEGs related to both anoikis and clinical features of PC. We then constructed a prognostic signature combining five of the 13 clinically relevant anoikis-related genes (*CKS2*, *CDC20*, *FMOD*, *CD38*, and *MSMB*), which had favorable performance for prognosis. A nomogram that combined Gleason score, T stage, and risk score was generated that could accurately predict patient survival. Furthermore, key hallmark gene sets related to DNA repair were differentially enriched between the high- and low-risk groups.

Anoikis is a specific form of apoptotic cell death that combats tumor metastasis. Compelling evidence has determined that this specific mode of apoptosis is relevant with survival in metastatic PC patients (Rennebeck et al., 2005a). The reactive stroma and EMT are involved in metastatic PC development, which is the structure basis for anoikis resistance. Accumulating evidence has implicated anoikis-related genes and pathways in the progression of various cancers, including lung cancer (Wang et al., 2022), nasopharyngeal carcinoma (Hao et al., 2020), and colorectal cancer (Takagi et al., 2020). However, the anoikis players in PC has not been fully understood. Herein, based on the expression of anoikis-related genes, two anoikis-related molecular subtypes were identified. Patients in cluster 1 were characterized by worse prognosis, higher sis, and advanced clinical features (higher Gleason score, higher nonsynonymous TMB, and more advanced N stage). Stemness is always applied to assess the degree of similarity between tumor and stem cells (Saba et al., 2021). Tumor development has been attributed to progenitor-like and stem cell characteristics (Malta et al., 2018). Tumors with cancer stem cell properties have a higher probability of aggressive migration or distant metastasis (Celià-Terrassa and Jolly, 2020). Therefore, we speculate that higher sis and advanced clinical features may be responsible for the poor prognosis of cluster 1. Moreover, the tumor microenvironment plays a significant role in survival and prognosis (Hinshaw and Shevde, 2019). Diverse immune cells in the tumor microenvironment are associated with PC development and immunotherapy outcomes (Kwon et al., 2021). Differential immune cells, such as M0 and M2 macrophages, had higher infiltration levels than other differential immune cells between subtypes and may be key immune cells between subtypes. Meanwhile, decreased expression of most immune checkpoint genes, such as *PD-1*, *PD-L1*, and *CTLA4*, was observed in cluster1, implying an association between subtype and immunotherapy outcomes, and patients in cluster 1 may benefit more from immune checkpoint therapy. Taken together, these data suggest that anoikis-related genes may affect the disease risk in different subtypes by affecting stemness, clinical features, and the tumor microenvironment. However, it is no longer convincing to use BULK data to discuss immune infiltration status, and the results of this study will be further explored in other types of datasets.

Several anoikis-related gene signatures have been developed to assess tumor progression and prognosis in patients with diverse cancers, such as endometrial carcinoma (Chen et al., 2021), low-grade gliomas (Zhao et al., 2022), and head and neck squamous cell carcinoma (Chi et al., 2022). In the present study, the anoikis-related gene signature comprised five genes (*CKS2*, *CDC20*, *FMOD*, *CD38*, and *MSMB*) and exerted a high prognostic value in both the training and independent PC validation cohorts. With regard to the critical role of anoikis in metastasis, we verified the predictive value of the identified anoikis-related gene signature in metastatic PC. Based on GSE211448 database, the risk score of each sample was calculated based on the expression of five anoikis-related genes. Results showed that the risk score was significantly different between metastatic and primary PC group, implying the potential predictive value of anoikis-related gene signature in the metastatic PC. CKS2 is a key regulator of the cell cycle and highly expressed in many cancers (You et al., 2015; Yu et al., 2015). Wang et al. revealed that CKS2 is associated with the recurrence and prognosis of PC (Wang et al., 2020). CDC20 is also a regulator of cell cycle checkpoints, and its increased expression is related to poor pathological features and poor prognosis in a variety of human cancers (Moura et al., 2014; Zhang et al., 2019; Jeong et al., 2022). In metastatic PC, CDC20 is also highly expressed and related to poor DFS (Dai et al., 2021). FMOD, a small leucine-rich proteoglycan in the ECM, has been implicated in the pathogenesis of several pathological conditions, including tumors (Al-Qattan and Al-Qattan, 2018). FMOD shows increased expression in PC tissues and may be used as a potential biomarker for PC (Bettin et al., 2016). CD38, a druggable ectoenzyme, has been shown to be expressed on diverse prostate tumor-infiltrating immune cells (TIICs), and the CD38^+^ TIIC density is independently related to worse overall survival of patients with PC (Guo et al., 2021). MSMB is a major secretory product of prostate epithelial cells and plays a protective role in the suppression of PC (Haiman et al., 2013). MSMB has been suggested as a biomarker for the progression and recurrence of PC (Whitaker et al., 2010). Most of the five genes were confirmed to be differentially expressed in metastatic PC tissues, compared with primary PC. Our study also showed that these anoikis-related genes were related to PC prognosis, suggesting their usefulness as potential prognostic biomarkers (Bendifallah et al., 2016).

To better understand the possible regulatory mechanism underlying the different risks for PC recurrence, GSEA was performed and upregulated hallmark gene sets, such as DNA repair, were enriched in the high-risk samples. DNA repair is a complex pr ocess tightly linked to many types of human cancers considering that DNA repair defects have been associated with higher mutation rates, elevated genomic instability levels, and increased intratumoral heterogeneity (Gavande et al., 2016; Turgeon et al., 2018). Several studies have also demonstrated the effect of DNA repair defects on PC progression (Warner et al., 2019; Bryce et al., 2020). Based on our results, we speculate that anoikis-related genes may regulate PC development by affecting DNA repair.

## 5 Conclusion

Two related molecular subtypes of PC were identified, and cluster 1 had a poor prognosis, which was associated with higher stemness, advanced clinical features, and differential immune cell infiltration. A novel, clinically relevant five-anoikis-related gene signature was revealed as a powerful prognostic biomarker for PC. Our findings expand our knowledge of anoikis in PC and contribute to a more accurate prognostic evaluation of patients with PC.

## Data Availability

The raw data supporting the conclusion of this article will be made available by the authors, without undue reservation.
